# *Rickettsia aeschlimannii* Infection in a Man, Greece

**DOI:** 10.3201/eid1907.130232

**Published:** 2013-07

**Authors:** Antonis Germanakis, Dimosthenis Chochlakis, Emmanouil Angelakis, Yannis Tselentis, Anna Psaroulaki

**Affiliations:** General Hospital of Agios Nikolaos, Crete, Greece (A. Germanakis);; Regional Laboratory of Public Health, Heraklion-Crete, Greece (D. Chochlakis, Y. Tselentis);; University of Crete Medical School, Heraklion, Greece (Emmanouil Angelakis, Anna Psaroulaki);; Aix-Marseille University School of Medicine, Marseille, France (E. Angelakis)

**Keywords:** *Rickettsia aeschlimannii*, bacteria, coccobacillus, zoonoses, vector-borne infections, ticks, spotted fever group, Greece, Rickettsia

**To the Editor:** In Greece, 6 spotted fever group (SFG) *Rickettsia* species have been detected in ticks: *Rickettsia conorii, R. massiliae, R. aeschlimannii*, *R. sibirica mongolitimonae*, *R. slovaca*, and *R. rhipicephali* (*1*). SFG species present characteristic clinical signs, including high fever, headache, and maculopapular rash; an inoculation eschar at the tick bite site is characteristic of some, but not all, SFG rickettsioses. Symptoms during the early stages of illness are nonspecific, and diagnosis is a challenge for physicians who are not familiar with rickettsial diseases. So far, 2 SFG *Rickettsia* species have been implicated in human disease in Greece: Mediterranean spotted fever caused by *R. conorii* (*2*), and lymphagitis-associated rickettsiosis (LAR) caused by *R. sibirica mongolitimonae* (*3*). We report a rickettsiosis case in a man on the island of Crete, Greece caused by a third *Rickettsia* species belonging to the SFG, *R. aeschlimannii*.

During June 2010, a 70-year-old man residing in an agricultural area of eastern Crete was admitted to the emergency unit of General Hospital of Agios Nikolaos for evaluation of a reddish, painless papule on the anterior surface of his left arm. The papule was 2 cm in diameter, and was surrounded by a less reddened infiltrated area 8 cm in diameter (Figure). The area was without tenderness or pruritus. At the center of the papule, which was cyanotic, the presence of a tick was recorded, and the tick was removed carefully in its entirety. The patient was afebrile and reported no other symptoms. The papule had developed within few hours, although 5 days previously, the patient had noticed a dark colored nodule on his left arm but paid no attention to it. The patient reported that rabbits were bred and goats and sheep grazed at close proximity to his residence.

**Figure:**
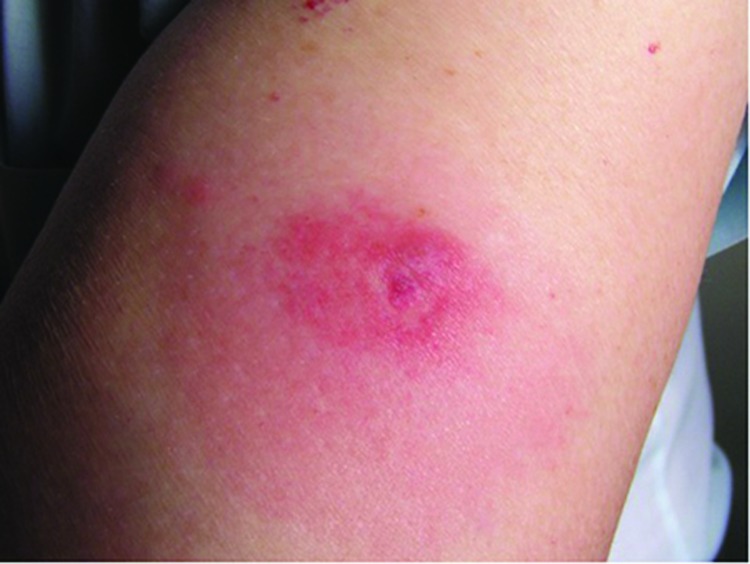
Papule on the anterior surface of the left arm of a 70-year-old man, Crete, Greece. The papule was surrounded by an infiltrated area without tenderness or pruritus. A tick was found in the center of the papule and carefully removed in its entirety.

 Serum and whole blood samples were drawn, and a local skin biopsy was performed from the center of the skin lesion. Laboratory tests revealed a high level of C-reactive protein, microscopic hematuria, and a leukocyte count of 6.01 × 10^9^ cells/L. Hepatic enzymes alanine transaminase and aspartate aminotransferase were within normal ranges. The patient was treated with doxycycline, 100 mg twice daily for 7 days; he did not develop further symptoms, and the skin lesion healed without ulceration.

All samples were sent to the Laboratory of Clinical Bacteriology, Parasitology, Zoonoses and Geographic Medicine at the University of Crete for further testing. The tick was identified as *Rhipicephalus turanicus* by using standard taxonomic keys (*4*). IgG and IgM titers reactive to SFG rickettsiae antigens were determined by an immunofluorescence antibody assay as described by the manufacturer (bioMérieux, Marcy l’Etoile, France). 

Twenty days after initial assessment and treatment, convalescent-phase blood samples were drawn for serum and whole blood analysis. Titers against *R. conorii* were detected in both the initial samples (IgM 1/100, IgG 1/60) and the convalescent-phase samples (IgM 1/100, IgG 1/120). DNA was extracted from the blood samples, the skin biopsy, and the tick by using a QIAamp Tissue Kit (QIAGEN, Courtaboeuf, France ) and used as a template in previously described PCR assays by using primers RpCS 877p-RpCS 1258n and Rr19070p-Rr190602n, targeting a 381-bp portion of the *gltA* and a 532-bp portion of the *ompA* genes of *Rickettsia* spp. (*5*). The whole blood drawn in the hospital, the skin biopsy, and the tick were positive for both genes. However, the convalescent-phase blood sample was negative. 

PCR products were purified by using the QIAquick Spin PCR Purification Kit (QIAGEN) and sequenced (Bioanalytica–Genotype, Athens, Greece) according to the manufacturer’s instructions. Sequences obtained shared 100% similarity to the corresponding fragment of the genome of *R. eschlimannii* (*gltA*: JF803904; *ompA*: JF803906). All samples were cultured in human embryonic lung fibroblasts as described (*6*). After 4 weeks, no bacteria were isolated. 

We report a human case of *R. eschlimannii* infection in Crete, Greece. Our finding was confirmed by molecular methods. However, we were not able to cultivate *R. aeschlimannii* from samples collected. This result suggests that living microorganisms may have died before testing or that only DNA, but no living organism, was present in the samples. *R. aeschlimannii* was first isolated from *Hyalomma marginatum* ticks from Morocco (*7*). In Europe, *R. aeschlimannii* has also been found in ticks from Germany, Russia, Italy, France, Croatia, Portugal, and Spain (*8*). In Greece, *R. aeschlimannii* has been detected in *H. anatolicum excavatum* ticks collected from sheep (*1*). The tick removed from this patient was *Rh. turanicus*, a species that has been reported in Spain to be infected with *R. aeschlimannii* (*9*). 

The first human case of *R. aeschlimannii* infection was identified in a patient who had fever, rash, and an eschar after travel in Morocco (*10*). *R. aeschlimannii* infections in humans have been previously confirmed in South Africa, in Algeria, and in Tunisia (*8*). To our knowledge, human cases of *R. aeschlimannii* infection have not been reported in Europe. Our results emphasize that ticks should be considered as potential vectors for rickettsial infections in humans. We recommend that when one species or serotype of tick-transmitted *Rickettsia* is identified in an area, physicians be informed through established clinical or public health channels of the potential pathogen, its manifestations, and recommended treatments for humans. 
